# Microbiological diagnostic procedures for respiratory cystic fibrosis samples in Spain: towards standard of care practices

**DOI:** 10.1186/s12866-014-0335-y

**Published:** 2014-12-24

**Authors:** Juan de Dios Caballero, Rosa del Campo, Marta Tato, Elia Gómez G de la Pedrosa, Marta Cobo, Carla López-Causapé, Enrique Gómez-Mampaso, Antonio Oliver, Rafael Cantón, Spanish Network for Cystic Fibrosis Microbiology Laboratories

**Affiliations:** Servicio de Microbiología, Hospital Universitario Ramón y Cajal and Instituto Ramón y Cajal de Investigaciones Sanitarias (IRYCIS), Madrid, 28034 Spain; Red Española de Investigación en Patología Infecciosa (REIPI), Madrid, Spain; Servicio de Microbiología y Unidad de Investigación, Hospital Universitario Son Espases, Instituto de Investigación Sanitaria de Palma (IdISPa), Palma de Mallorca, Spain

**Keywords:** Cystic fibrosis, Microbiology, Laboratory procedures, Respiratory samples, Consensus guidelines

## Abstract

**Background:**

The microbiological procedures for cystic fibrosis (CF) samples of 17 participating Spanish centers were examined to verify their compliance with current international and national guidelines and to implement the best standards of care for microbiology practices. A 47-item questionnaire covering different CF microbiology aspects was sent to participant laboratories. Telephone interviews were performed when necessary. Data about samples processing for bacteria, mycobacteria and fungi were collected.

**Results:**

Gene sequencing (71%), MALDI-TOF (59%) or both (94%) were available for most laboratories. Susceptibility testing was performed by automated microdilution systems (94%) and manual diffusion methods (59%). However, a low use of selective media for *Staphylococcus aureus* (59%) and *Burkholderia cepacia* complex (71%), and of epidemiological typing methods (41%) was reported.

**Conclusions:**

Most Spanish laboratories are in agreement with consensus guidelines for the processing of CF respiratory samples, but need to improve in the use of specific selective media and typing methods for epidemiologic studies.

**Electronic supplementary material:**

The online version of this article (doi:10.1186/s12866-014-0335-y) contains supplementary material, which is available to authorized users.

## Background

Cystic fibrosis (CF) disease is produced by mutations in the CF transmembrane conductance regulator gene (CFTR). Altered CFTR leads to the production of viscous secretions in respiratory airways that cannot be cleared by the mucociliary system and patients get chronically colonized by different microorganisms (bacteria, mycobacteria and fungi) which cause inflammation, progressive lung destruction and, finally, death by respiratory failure [1].

Microbiological diagnosis of CF has evolved far beyond the isolation and identification of classic pathogens such as *Pseudomonas aeruginosa* and *Staphylococcus aureus* [2]. Early diagnosis of CF disease and better strategies of patient management have substantially increased patients’ life expectancy with a subsequent impact on CF pathogens epidemiology [2,3]. Continuous follow-up of microbial colonization represents a challenge to clinical laboratories for its complexity and has become a standard of care in patient management. Recommendations for the CF microbiology laboratory management have been included in the European Cystic Fibrosis Society (ECFS) guidelines, as part of the framework of a specialized CF center [4,5].

High antibiotic pressure and the special environment of the CF lung allow the establishment of multi-drug resistant bacteria that require special techniques for their isolation and/or identification, such as *Burkholderia cepacia* complex (BCC), other non-fermenting Gram negative rods (NFGNR) and nontuberculous-mycobacteria (NTM) [2,3]. In addition, commonly isolated pathogens such as *P. aeruginosa* or *S. aureus* can exhibit altered phenotypic characteristics as a result of time-dependent adaptive phenotypic changes to the CF lung, including small-colony variants (SCVs) and hyper-mutable and mucoid strains variants. Correct diagnosis of these phenotypic variants is difficult and has clinical relevance as there is growing evidence that correlates them with multi-drug resistance, persistency phenomena and poorer lung function [6-9]. Moreover, accurate isolation, identification and susceptibility testing of CF pathogens are critical for ensuring appropriate treatment and implementation of infection control measures, and also for improving our understanding of CF microbiology [10-14].

Laboratories working with CF samples need special procedures and installations as well as specialized microbiologists to provide a correct patient assessment for clinicians [4,10-14]. Adherence to consensus guidelines is also important for laboratories to obtain comparable results and for their adaptation to the best standards of care in CF patients [4,10,14]. The aim of this work was to compile information about the microbiological procedures of the Spanish Hospitals with CF Units in order to assess their compliance with recent consensus guidelines and to implement general recommendations for CF samples processing.

## Results

### Hospitals and laboratories

Seventeen hospital microbiology laboratories covering all the Spanish territory were requested to participate by answering to our questionnaire (Table 1) and all of them agreed. These centers, which are reference CF Units in their corresponding geographic areas, were selected due to their collaboration with us in another multicenter study focused on CF microbial colonization patterns. Although the precise number of CF patients in our country is unknown since no national patient registry exists, centers included in this survey attend to the majority of the Spanish CF population. The total population attended by these hospitals is approximately 7,150,000 people (mean 420,502). The total number of hospital beds is 15,183 (mean 893) and 1,037 (mean 61) for intensive care units (ICU). The number of CF patients attended by these centers is 2,315 (Table 1), which represents 75% of the CF Spanish population according with the last ECFS report [15].

**Table 1:** **Characteristics of the Spanish hospitals participating in the study**

**Geographical area**	**Hospital name**	**Population attended**	**Number of beds**		**Number of CF patients**
			**Total**	**ICU**	
Madrid	Ramón y Cajal	550,000	1,100	60	150
	12 de Octubre	480,252	1,300	96	208
	La Paz	500,000	1,200	67	151
	La Princesa	320,000	564	22	87
	Niño Jesús	90,000	180	14	85
Asturias	Central de Asturias	500,000	1,000	50	51
Basque Country	Cruces	384,000	865	24	209
Catalonia	Parc Tauli	394,000	714	30	75
	San Joan de Déu	200,000	300	44	55
	Vall d’Hebrón	453,196	1,146	182	175
Balearic Islands	Son Espases	330,000	1,020	107	40
Valencian Community	La Fe	198,889	980	100	344
	Clínico de Valencia	350,000	500	20	40
Region of Murcia	Virgen de la Arrixaca	254,000	900	58	130
Andalusia	Vigen del Rocío	820,904	1,367	62	360
	Hospital Regional de Málaga	623,301	1,147	61	105
Canary Islands	Nuestra Señora de la Candelaria	700,000	900	40	50
**TOTAL**		**7,148,542**	**15,183**	**1,037**	**2,315**

**Figure 1:**
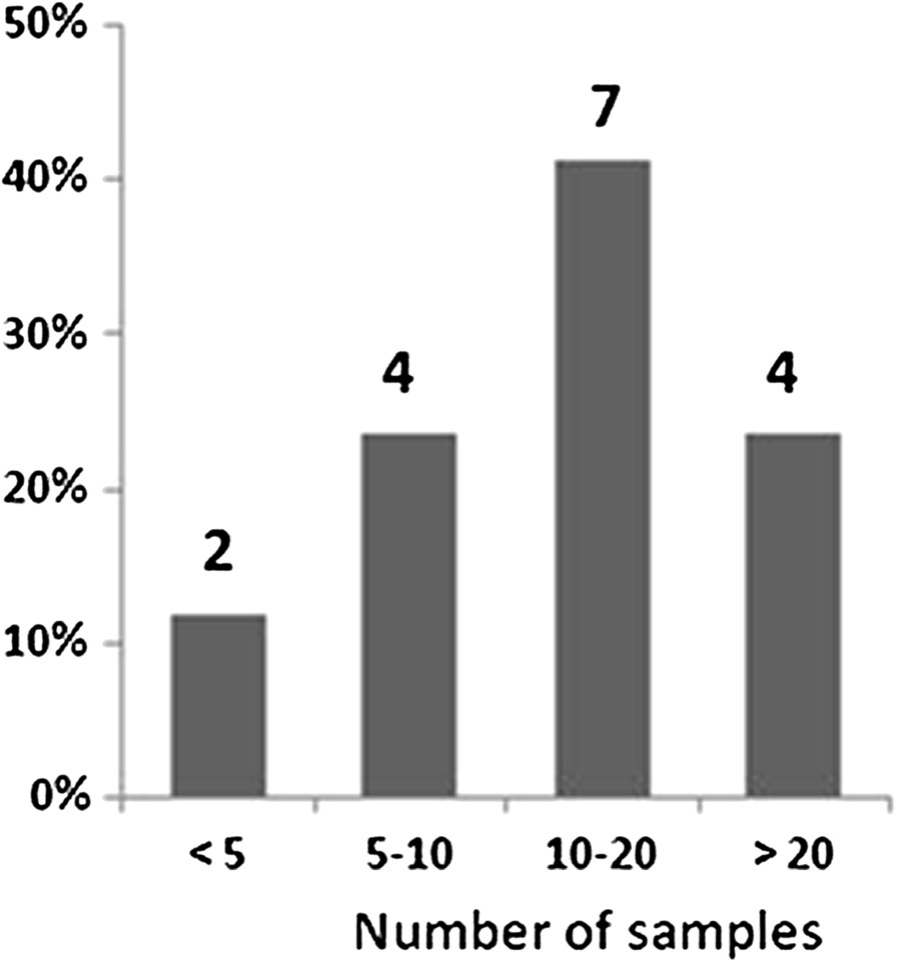
**CF samples processed weekly by participant laboratories.**

The number of CF samples processed weekly by each laboratory varies from 10 to 20 in the majority of cases (n = 7, 41%, Figure 1). Only 5 laboratories (29.4%) have a CF section exclusively dedicated for the CF samples processing, although in all cases the final report to clinicians was under the responsibility of a clinical microbiologist. Sixteen laboratories (94%) have written protocols for processing CF samples. More than half are certified by a Quality Management System (n = 10, 58.8%), the most commonly implemented being ISO9001 (n = 8; 80%) followed by other national or regional systems (n = 2; 20%). All laboratories follow a periodical external quality control, which is performed by the Spanish Society of Infectious Diseases and Clinical Microbiology (SEIMC, http://www.seimc.org) in all cases.

### CF samples processing

Spontaneous sputum was reported as a very frequent or a frequent sample in 94% and 6% of the centers and nasopharyngeal swabs in 29% and 41%, respectively. Induced sputum, bronchoalveolar lavage and bronchoaspirate were very infrequent or absent in 77%, 65% and 65% of the centers, respectively.

Initial homogenization of sputum for bacterial and fungal culture was performed by nearly all laboratories (n = 16, 94%), N-acetylcysteine (NAC) and dithiothreitol being the most common chemical agents used (n = 11, 65% and n = 4, 23.5%; respectively). Samples pretreatment for NTM culture was reported by 13 laboratories; the combination of NAC plus 2% NaOH being the preferred option (Kubica-Krasnow method; n = 11), followed by the combination of 3% sodium lauryl sulphate plus 1% NaOH (Tacquet-Tison method; n = 2).

Quantitative culture for bacterial pathogens was routinely carried out by only 14 laboratories (82%), using calibrated loops (n = 7) or serial dilutions plus whole plate seeding (n = 7). Fungal CF colonizers were cultured routinely in 13 centers and on the clinician’s request in 4. NTM culture of CF samples was reported in 15 (88%) laboratories. Anaerobic bacteria in CF samples were never considered as relevant in 14 centers and cultured in 3 laboratories only under clinician’s request.

### Isolation, identification and antibiotic susceptibility testing of CF pathogens

The use of culture media for bacterial, mycobacterial and fungal pathogens is shown in Table 2. Columbia blood agar, chocolate agar and MacConkey agar were universally used but selective media for *S. aureus* and BCC were not present in all laboratories (59% and 71%, respectively). For culturing NTM, automated systems with liquid enrichment media were the most frequently used (n = 14; 93%). Sabouraud-chloramphenicol agar was universally used for culturing fungi.

**Table 2:** **Culture media used for the isolation of CF pathogens**

**Culture medium**	**N° of laboratories using media (%)**
**Bacteria**	17 (100)
• Columbia blood agar^*a*^	17 (100)
• Chocolate agar^*b*^	17 (100)
• MacConkey agar	17 (100)
**Selective media for:**	
• *S. aureus* ^*c*^	10 (59)
• *P. aeruginosa*	0 (0)
• BCC	12 (71)
**Mycobacteria:** ^***d***^	15 (88)
• Lowenstein-Jensen^*e*^	8 (53)
• Coletsos	3 (20)
• Liquid enrichment media	14 (93)
**Fungi:** ^***f***^	17 (100)
• Sabouraud agar^*g*^	17 (100)

^*a*^Blood agar alone (n = 16) or supplemented with nalidixic acid (n = 3).

^*b*^Chocolate agar alone (n = 16) or supplemented with bacitracin plus colistin (n = 3) or with bacitracin plus amphotericin B (n = 1).

^*c*^Mannitol salt agar (n = 7), Columbia blood agar plus nalidixic acid (n = 3) and chromogenic agar (n = 2).

^*d*^15 out of 17 laboratories answered this question.

^*e*^Lowenstein-Jensen alone (n = 2) or supplemented with antibiotics (n = 6) or piruvate (n = 1).

^*f*^Both yeasts and filamentous fungi.

^*g*^Sabouraud-Chloramphenicol agar alone (n = 13) or supplemented with gentamicin (n = 2) or actidione (n = 1).

**BCC:** *Burkholderia cepacia* complex.

Identification techniques used by the clinical laboratories are shown in Table 3. Traditional procedures, including biochemical tests or microscopy (for filamentous fungi), were widely used for determining bacterial and fungal species. The main techniques for identification of NTM were hybridization assays (n = 13) followed by biochemical tests (n = 9). For in depth identification of bacteria, mycobacteria and fungi, use of gene sequencing was reported in 12 (71%), 9 (53%) and 10 (59%) laboratories, respectively. The corresponding figures for mass spectrometry were 10 (59%), 9 (53%) and 8 (47%), respectively. Nearly all centers (n = 16, 94%) had at least one of both techniques.

**Table 3:** **Identification techniques used in CF clinical microbiology laboratories no. (%)**

	**Bacteria**	**Mycobacteria**	**Yeasts**	**Filamentous fungi**
**BCT**	17 (100)	9 (53)	8 (47)	9 (53)
**Agglutination assays**	17 (100)	-	-	-
**SFT**	-	-	11 (65)	-
**Microscopy**	-	-	-	15 (88)
**MALDI-TOF**	10 (59)	9 (53)	8 (47)	8 (47)
**PCR + sequencing**				
rDNA^*a*^	11 (65)^*c*^	8 (47)^*d*^	6 (35)^*e*^	6 (35)^*f*^
ITS region	-	-	7 (41)^*e*^	8 (47)^*f*^
Others^*b*^	5 (29)^*c*^	-	-	-
**Hybridization assays**	-	14 (82)	-	-
**RFLPs**	-	3 (18)^*d*^	-	-

**BCT:** Biochemical tests; **SFT:** serum filamentation tests. **RFLPs:** Restriction Fragment Length Polymorphisms.

^*a*^16S rDNA in the case of bacteria and 18S rDNA in the case of fungi.

^*b*^ *recA* gene, *hsp65* gene, etc.

^*c*^4 centers reported the use of >1 technique.

^*d*^3 centers reported the use of both techniques.

^*e, f*^4 centers reported the use of both techniques.

Antibiotic susceptibility testing of bacterial CF pathogens was performed by all participant laboratories. Most of them reported the use of automated microdilution systems (n = 16) and agar diffusion techniques, either disk diffusion (n = 2) or gradient strips (Etest®, n = 4), or both (n = 10). Automated systems were MicroScan (n = 8), VITEK2 (n = 3) or both (n = 5).

Antifungal susceptibility is routinely evaluated in 2 (12%) centers and under special conditions in 8 (47%) which include clinician’s request (n = 4), clinically significant isolates (n = 2), treatment refractory cases (n = 2), isolation of *Scedosporium* spp. (n = 1) and pre-transplant patient status (n = 1). Susceptibility assays are performed by broth microdilution (n = 3), agar diffusion with antifungal gradient strips (Etest®, n = 3) or both (n = 4).

Most laboratories (n = 11) report culture results in 3–5 days and the rest in 1–3 days (n = 5) or >5 days (n = 1). Information sent to clinicians of bacterial culture is summarized in Table 4.

**Table 4:** **Information reported to clinicians for CF samples**

**Information included in the laboratory report**	**No. of centers (%)**
Isolated species	17 (100)
Antibiotic susceptibility	17 (100)
Use of different MIC interpretation for inhaled antibiotics^*a*^	5 (29)
Total count of microorganisms	3 (18)
Individual count of each species	11 (65)
*P. aeruginosa* morphotype	16 (94)
*P. aeruginosa* hypermutable trait	4 (24)
*S. aureus* SCVs	4 (24)

**MICs:** Minimal inhibitory concentrations; **SCVs:** Small-colony variants.

^*a*^Inhaled tobramycin breakpoints for *P. aeruginosa*: Susceptible (≤64 μg/mL) or resistant (>64 μg/mL).

Clonal relationships between strains for epidemiological studies were performed in 7 (41%) laboratories, the pulse field gel electrophoresis (PFGE) being the main technique used, alone (n = 3) or in combination with multi-locus sequence typing (MLST) (n = 4).

## Discussion

A correct processing of CF samples is critical to identify the maximum number of potential pathogens in the respiratory airways. Incorrect results in the isolation, identification and susceptibility testing of CF organisms have negative consequences in the patient’s clinical management and quality of life, and can affect the whole CF community by delaying the implementation of appropriate infection control measures to prevent patient-to-patient transmission [10-14,16-18]. Pathogens with known clinical and epidemiological importance, such as *P. aeruginosa*, *S. aureus* or BCC, could be misidentified due to their phenotypic variation or to the limitations of the classical culture techniques with these organisms [6,18]. Less known pathogens, such as non-*P. aeruginosa* NFGNR, filamentous fungi, certain yeasts and NTM, could also be missed. Standardized microbiological procedures could help to avoid this situation and to better understand its clinical and epidemiological importance. The present work is an overview of the proceedings of the main CF Spanish laboratories that might be used to improve the clinical microbiological procedures in line with current guidelines and with the recommendations of standards of care from the European Cystic Fibrosis Society [4,5,10,14].

Culturing CF samples is one of the most labor-intensive procedures of the Microbiology Laboratory [18]. It requires the use of different media and the identification and susceptibility testing of multiple isolates per patient, employing techniques that are more complex than those used for non-CF samples, and with difficult to interpret results. In our study, only 5 laboratories have an individual section within the Microbiology Department dedicated to CF. Even thought a clinical microbiology specialist is always responsible for the results, potentially assuring better compliance with the currently recommended framework for CF centers [4], the presence of experienced personnel is critical to recognize and isolate all the specific CF pathogens and their phenotypic variants [4]. Prolonged incubation times are also needed for the isolation of these variants and of BCC species [10,14,18]. However, most of the laboratories reported culture results in less than 5 days, which might be insufficient for these pathogens. A positive result is that all laboratories participate in external quality assurance programs which are crucial to evaluate and continuously improve the quality of the laboratory performance [4]. Unfortunately, there are no specific quality assurance programs for CF in our country. The implementation of these programs, using multiple CF pathogens and phenotypic variants, would be desirable for a correct evaluation of the CF laboratory [17].

Almost all laboratories homogenize sputum with mucolytic agents as currently recommended for the more viscous CF airways secretions, in which microorganisms grow as biofilm-like microcolonies [14,17]. However, quantitative culture using serial sample dilutions were not performed in all centers. This explains why few laboratories report individual bacteria counts (68%) and even fewer total bacterial load (18%). Although the clinical value of quantitative culture is controversial, it is a useful practice as it permits an efficient separation of different CF pathogens and their phenotypic variants even when present in low densities, preventing the overgrowth of *P. aeruginosa*. Moreover, it can also serve for monitoring treatment efficacy [14,17].

While all laboratories report the presence of mucoid *P. aeruginosa* morphotype to clinicians, few of them (24%) inform about hyper-mutable traits or SCVs of *P. aeruginosa* or *S. aureus* isolates. These variants could modify treatment strategies as they are related to antibiotic resistance and persistency, so informing clinicians about their presence could be clinically and epidemiologically important [6-9]. Performing quantitative cultures and using selective and chromogenic media, along with prolonged incubation times can help in the detection of SCVs. There are several methods described to identify hyper-mutable strains, although the observation of microcolonies within the inhibition zones when using disk diffusion and/or MIC strips for susceptibility testing is probably the easiest method for this objective [10,14,19].

American, European and Spanish CF guidelines strongly recommend selective media for the isolation of *S. aureus*, *P. aeruginosa*, BCC and *Haemophilus influenzae* [10,14,20]. While all laboratories use MacConkey and chocolate agars for the *P. aeruginosa* and *H. influenzae* isolation, not all of them use specific selective media for *S. aureus* (59%) and BCC (71%). These rates are considerably low when compared with the corresponding figures in Germany (69% and 91%, respectively) and USA (82% and 99%, respectively) [12,13]. Lack of these media is associated with lower isolation rates of these organisms, which are difficult to identify in the CF context and that can be easily obscured by the overgrowth of *P. aeruginosa*. Missing pathogens like BCC or methicillin-resistant *S. aureus* (MRSA) is especially worrisome since it could have an impact not only at patient level but affecting also the whole CF community by patient-to-patient transmission [10,11,18]. A very positive result of the study is that nearly all laboratories have molecular and/or mass spectrometry assays for the identification of CF pathogens. Conventional biochemical tests, including those in automated systems, often give false identification results for NFGNR and phenotypic variants of *P. aeruginosa*, and are unreliable for identifying single species of the BCC [13,17,18,21]. PCR based techniques are recommended for the identification of these pathogens and can serve also for BCC [14]. However, mass spectrometry is a rapid, cheaper and a reliable alternative to PCR for these organisms, and is also a promising tool for mycobacteria and fungi [22-25].

Another positive result of the study is the use in nearly all laboratories of agar diffusion tests in addition to automated microdilution systems for antimicrobial susceptibility testing. Use of both techniques is especially relevant. Automated microdilution systems alone are not recommended for *P. aeruginosa* due to unacceptable major (false resistance) and very major (false susceptibility) errors [10,14]. On the contrary, disk diffusion and gradient strips correlate better with reference methods and have the advantages of detecting hyper-mutable variants and, in the case of gradient strips, the use of a wider range of concentrations allows to the application of proposed breakpoints for inhaled therapy [14,26].

*S. aureus,* including MRSA, *P. aeruginosa* and BCC are pathogens with a proved spreading between CF patients but patient-to-patient transmission of *S. maltophilia*, *A. xylosoxidans* and NTM has also been suggested [10,27,28]. However, only 7 (41%) laboratories reported the use of PFGE or MLST techniques for routine epidemiologic studies and none of them reported the use of next generation sequencing procedures that are being increasingly introduced in clinical laboratories.

On the other hand, NTM prevalence has been rising during the last years and has been recently estimated as 10% of all CF patients, *Mycobacterium avium* complex and *M. abscessus* being the most commonly isolated [29]. The only recommendation for culturing NTM is an adequate pretreatment of CF samples to minimize culture contamination by *P. aeruginosa* [10,14]. Most Spanish laboratories perform the Kubica-Krasnow method with NAC plus 2% NaOH. Consensus guidelines recommend a second decontamination step with 5% oxalic acid to reduce contaminations with *P. aeruginosa* [10,14]. Recently, a more sensitive method using chlorhexidine has been described but it interferes with the automated liquid enrichment media culture systems [10,30].

Although CF consensus guidelines also recommend fungal cultures [4,10,14], no specific information is provided about the best procedures in this setting. While all Spanish laboratories use Sabouraud-Chloramphenicol agar, better results have been achieved using selective media for CF fungal colonizers, suggesting that the prevalence of these organisms has been probably underestimated in CF [31,32]. Protocols for culturing fungi are, therefore, needed to elucidate their possible role in CF pathogenesis.

In our study few laboratories cultured respiratory samples under anaerobic conditions. Currently, there are no specific recommendations for this practice in CF samples. However, recent microbiome studies suggested a possible role of these organisms, especially *Prevotella* spp. [2]. Since isolation, identification and susceptibility testing techniques for these organisms are difficult to perform, next generation systems and metagenomic tools would probably be the preferred option to study the role of these bacteria in CF [2].

## Conclusions

In summary, there are few studies regarding the assessment of microbiological procedures in CF samples and this is the first one performed in our country [12,13,17]. The results show that Spanish CF laboratories are generally in agreement with National, European and American consensus CF guidelines. However, certain procedures should be improved, such as creation of specific CF sections within the laboratory, inclusion of selective media for *S. aureus* and BCC and implementation of typing methods for epidemiologic studies. These actions will facilitate Spanish CF microbiology laboratories to be in line with recommended standards of care for Microbiology laboratories within the framework of European CF centers [4]. Moreover, this alignment will improve microbiological diagnosis and, subsequently, patient’s treatment, management and quality of life.

## Methods

A questionnaire for the participating laboratories was elaborated based on the recommendations for the microbiological diagnosis of bronchopulmonary infection in CF patients published by the Spanish Society of Clinical Microbiology and Infectious Diseases (SEIMC), by the Cystic Fibrosis Foundation^,^ and those included in National and International Guidelines for the management and treatment of CF patients [4,10,14,17]. It was a 47 questions document that was divided in three sections: i) general information about the hospital and the clinical microbiology laboratory; ii) general processing of CF samples and iii) isolation, identification and susceptibility testing techniques used for bacterial, mycobacterial and fungal CF pathogens (see Additional file 1). An online survey tool (SurveyMonkey Inc; Palo Alto, California, EEUU; http://www.surveymonkey.com) was used for sending the questionnaire to the participating laboratories and for analyzing the data obtained. Telephone interviews were performed when necessary to clarify specific answers to the questionnaire. This work was part of a multicenter study which was approved by Ramón y Cajal Hospital Clinical Research Ethical Committee (reference n° 341/12).

## Additional file

Additional file 1:
**Cystic Fibrosis Microbiology Questionnaire.** A PDF file of an English translated copy of the 47-intem questionnaire sent to the participant laboratories was included.
