# Supplementary material for: Microbiological diagnostic procedures for respiratory cystic fibrosis samples in Spain: towards standard of care practices
Source: BMC Microbiol. 2014 Dec 24;14:335. doi: 10.1186/s12866-014-0335-y (PMC4302700; doi:10.1186/s12866-014-0335-y)
Supplement: Additional file 1: — Cystic Fibrosis Microbiology Questionnaire. A PDF file of an English translated copy of the 47-intem questionnaire sent to the participant laboratories was included. [file 12866_2014_335_MOESM1_ESM.pdf]

INSTRUCTIONS FOR THE QUESTIONNAIRE

Type of questions

1. <u>Mandatory questions</u> (*): You must answer them to continue with the questionnaire.
2. <u>Single choice questions</u>: You can only choose an answer option. Answer options appear as circular bullets (●).
3. <u>Multiple choice questions</u>: You can choose more than one answer option. Answer options appear as square bullets (■).

Moving across the questionnaire: click on the following at the end of each page:

- Use ***Next*** to move forward in the survey
- Use ***Back*** to move back in the survey
- Use ***Exit*** if you want to finish the questionnaire
- Use ***Send*** to finish and send the survey

QUESTIONNAIRE: MICROBIOLOGICAL PROCEDURES FOR CYSTIC FIBROSIS (CF) SAMPLES

GENERAL INFORMATION

1. Personal information of the respondent*

Last name:

First name:

Hospital address:

E-mail:

Phone number:

2. Number of beds in your hospital*

Total number:

ICU number:

3. Total population attended in your center:*

4. Is your laboratory certified by a quality management system?*

- No
- Yes (specify the system, e.g. ISO 9001)

SECTION I: ORGANIZATION OF YOUR MICROBIOLOGY LABORATORY

5. Is there a specific CF section in your microbiology laboratory?*

- No
- Yes

6. Is there a specific person in charge of the CF microbiology?*

- No
- Yes

7. The person in charge of the CF section is:*

- ■ A clinical microbiologist
- ■ A medical resident
- ■ A nurse
- ■ A laboratory technician
- ■ Other (please specify)

8. If there is a physician in charge of the CF section, his/her responsibility is:*

- Rotational
- He/she is permanently in charge of the CF section

9. Apart from the person responsible of the CF section, is there any other staff?*

- No
- Yes, a laboratory technician/nurse full-time employed
- Yes, a laboratory technician/nurse part-time employed
- Yes, more than one laboratory technician/nurse. Please specify number

10. Is there any cystic fibrosis specific training program for your medical residents?*

- Yes (specify the duration)
- No

11. Do you have written protocols for the processing of cystic fibrosis samples?*

- Yes
- No

12. Your written protocols for the cystic fibrosis sample processing come from:*

- Expert consensus guidelines (e.g. SEIMC, ECFS, CFF)
- Standardized working protocols

13. Provide the number of samples processed weekly in your laboratory*

- Less than 5
- From 5 to 10
- From 10 to 20
- More than 20

14. Is your laboratory enrolled in an external quality control program?*

- No
- Yes

15. What is the name of the external quality control performed in your laboratory? (e.g. SEIMC, Spanish Society for Infectious Diseases and Clinical Microbiology)*

16. How often is the quality control performed?*

- Monthly
- Quarterly
- Biannual
- Annual

17. Do you perform periodical reports about the number and type of CF pathogens isolated and their susceptibility profile?*

- Yes, monthly
- Yes, quarterly
- Yes, biannually
- Yes, annually
- No

SECTION 2: CF SAMPLES PROCESSING

PART A: BACTERIAL PATHOGENS. This section refers only to CF bacterial pathogens (e.g. *Pseudomonas aeruginosa*, *Staphylococcus aureus*, etc). You will be able to answer on fungal and mycobacterial pathogens in parts B and C of this section. Please, answer accordingly.

18. Point out in this table the frequency of the different CF samples you received in your laboratory:*

	Never	Rarely	Frequently	Very frequently
Spontaneous sputum	•	•	•	•
Induced sputum	•	•	•	•
Bronchoalveolar lavage	•	•	•	•
Bronchoalveolar secretions	•	•	•	•
Nasopharyngeal lavage	•	•	•	•
Pharyngeal swab	•	•	•	•

19. Do you perform Gram stain in CF samples?*

- Yes, always
- Yes, rarely
- Never

20. What is the homogenization method of CF samples used in your laboratory?*

- ■ Sterile saline solution
- ■ N-acetylcysteine
- ■ Dithiothreitol
- ■ Sonication
- ■ We do not homogenize cystic fibrosis samples

21. Do you perform quantitative culture of CF samples?*

- Always
- Only under clinician's request
- Never

22. What is the method used for quantitative culture?*

- Serial dilutions
- Calibrated loops
- Others (please, specify):

23. Please, choose from the list below the media you use for culturing CF samples:*

- ■ Columbia blood agar
- ■ Columbia chocolated agar
- ■ Columbia chocolated agar supplemented with bacitracin and/or colistin
- ■ MacConkey agar
- ■ Mannitol salt agar
- ■ Chromogenic MRSA agar
- ■ Cetrimide agar
- ■ *Burkholderia cepacia* specific medium
- ■ Others (please, specify):

24. Culture results are reported to clinicians in a mean time of:*

- 1-3 days
- 3-5 days
- 5 days or more

25. Select from the list below the information you report to clinicians:*

- ■ Total bacterial count
- ■ Isolated species
- ■ Individual count of each isolate
- ■ Susceptibility profile
- ■ *P. aeruginosa* morphotype
- ■ *P. aeruginosa* hypermutable strains
- ■ *S. aureus* small-colony variants (SCVs)

26. Do you consider anaerobic microorganisms as clinically relevant in CF samples?*

- Yes, always
- No, never
- Yes, in some cases (please, specify):

27. Point out in the table the methods used in your laboratory for the identification of CF isolates and their frequency*

	Routinely	Occasionally	Not available
Biochemical tests	•	•	•
Agglutination assays	•	•	•
Mass spectrometry	•	•	•
rRNA sequencing	•	•	•
Other genes sequencing (e.g. *rec*A, *hsp*65)	•	•	•

28. Select the susceptibility testing methods you use from the list below*

- ■ Manual microdilution
- ■ Automated microdilution
- ■ Agar dilution
- ■ Agar disk diffusion
- ■ Gradient strips (Etest®)

29. Select from the list the automated microdilution systems used in your laboratory*

- MicroScan
- Vitek2
- Phoenix
- Others (please, specify):

30. Select from the list the antibiotics used for the susceptibility testing of *P. aeruginosa* isolates*

- Piperacillin-tazobactam
- Cefoxitin
- Ceftazidime
- Cefepime
- Aztreonam
- Imipenem
- Meropenem
- Gentamicin
- Tobramycin
- Amikacin
- Ciprofloxacin
- Levofloxacin
- Colistin
- Fosfomycin
- Others (please, specify)

31. Select from the list the antibiotics used for the susceptibility testing of *S. aureus* isolates*

- Penicillin G
- Ampicillin
- Amoxicillin-clavulanate
- Cefazolin
- Oxacillin
- Cefoxitin
- Gentamicin
- Amikacin
- Erythromycin
- Clindamycin
- Ciprofloxacin
- Levofloxacin
- Co-trimoxazole
- Vancomycin
- Fosfomycin
- Linezolid

32. Is there any explanatory remark in the susceptibility testing results about the MICs of inhaled antibiotics?*

- No
- Yes (please, specify):

33. Do you apply and inform the proposed clinical breakpoints for inhaled antibiotics?*

- Yes
- No

34. Please, select from the list the techniques used for epidemiological studies*

- We don't perform epidemiological studies
- Pulse Field Gel Electrophoresis (PFGE)
- Multi-Locus Sequence Typing (MLST)
- Ribotyping
- Others (specify):

PART B: FUNGAL PATHOGENS. This section refers only to the culture, identification and susceptibility testing of yeasts and filamentous fungi isolated from CF patients. Please, respond accordingly.

35. Do you perform fungal culture in cystic fibrosis samples?*

- Yes
- No

36. In your opinion, the isolation of fungal pathogens from CF samples is:*

- Very relevant for the patient
- Relevant for the patient
- Not relevant for the patient
- Depends on the patient (please, specify):

37. Choose from the list below the media you use for fungal culture in CF samples:*

- ■ Sabouraud-cloramphenicol agar
- ■ Sabouraud-cloramphenicol- cycloheximide agar
- ■ Erythritol chloramphenicol agar
- ■ Others (please, specify):

38. Point out in the table the methods used for the identification of yeasts and their frequency *

	Routinely	Occasionally	Not available
Biochemical tests	•	•	•
Auxonogram	•	•	•
Serum filamentation test	•	•	•
ITS sequencing	•	•	•
18S rRNA sequencing	•	•	•
Mass spectrometry	•	•	•

39. Point out in the table the methods used for the identification of filamentous fungi and their frequency *

	Routinely	Occasionally	Not available
Biochemical tests	•	•	•
Lactophenol blue staining	•	•	•
ITS sequencing	•	•	•
18S rRNA sequencing	•	•	•
Mass spectrometry	•	•	•

40. Is susceptibility testing of fungal CF pathogens routinely performed?

- No, never
- Yes, frequently
- Yes, in some cases (please, specify)

41. What methods are used for antifungal susceptibility testing?

- ■ Microdilution
- ■ Agar gradient strips
- ■ Agar dilution

PART C: MYCOBACTERIAL PATHOGENS. This section refers only to the culture, identification and susceptibility testing of non-tuberculous mycobacteria (NTM) isolated from CF patients. Please, respond accordingly.

42. Do you perform NTM culture in cystic fibrosis samples?*

- Yes.
- No.

43. Select from the list the NTM species that, in your opinion, are clinically relevant:*

- ■ *Mycobacterium avium.*
- ■ *M. intracelullare.*
- ■ *M. chelonae.*
- ■ *M. abscessus.*
- ■ *M. fortuitum.*
- ■ Others (please, specify):
- ■ We don't consider NTMs as clinically relevant.

44. What sputum decontamination method is used previously to NTM culture?*

- Kubica-Krasnow method (N-acetyl-cysteine + 2% NaOH).
- Taquet-Tison method (sodium lauryl sulfate + NaOH).
- Others (please, specify):

45. Select from the list the culture media used for the isolation of NTM:*

- ■ Coletsos.
- ■ Lowenstein-Jensen.
- ■ Middlebrock.
- ■ Liquid enrichment media.
- ■ Others (please, specify):

46. Do you perform any NTM specific staining of CF samples?*

- Auramine fluorescent staining.
- Ziehl-Neelsen staining.
- Others (please specify).
- We don't perform any NTM specific staining.

47. Point out in the table the methods used and their frequency for the identification of NTM*

	Routinely	Occasionally	Not available
Biochemical tests	●	●	●
Specific probe hybridization	●	●	●
Restriction assays (e.g. PCR + RFLPs *hsp*65)	●	●	●
Gene sequencing (e.g. 16S rRNA, *hsp*65, etc)	●	●	●
Mass spectrometry	●	●	●
